# Melt Crystallization Behavior and Crystalline Morphology of Polylactide/Poly(ε-caprolactone) Blends Compatibilized by Lactide-Caprolactone Copolymer

**DOI:** 10.3390/polym10111181

**Published:** 2018-10-24

**Authors:** Chunmei Zhang, Qiaofeng Lan, Tianliang Zhai, Shengqiang Nie, Jun Luo, Wei Yan

**Affiliations:** 1 College of Chemistry and Materials Engineering, Guiyang University, Guiyang 550005, China; zhangzhang_87@126.com (C.Z.); nieshq1987@163.com (S.N.); luojun_gyu@sina.com (J.L.); lrasyw@163.com (W.Y.); 2 Biomaterials Research Center, School of Biomedical Engineering, Southern Medical University, Guangzhou 510515, China; 3 Guizhou Building Material Quality Supervision Testing Center, Guiyang 550000, China; ztl.mzl@163.com

**Keywords:** polylactide, poly(ε-caprolactone), compatibilization, melt crystallization

## Abstract

Lactide-Caprolactone copolymer (LACL) was added to a Polylactide/Poly(ε-caprolactone) (PLA/PCL) blend as a compatibilizer through solution mixing and the casting method. The melt crystallization behavior and crystalline morphology of PLA, PLA/PCL, and PLA/PCL/LACL were investigated using differential scanning calorimeter (DSC) and polarized optical microscopy (POM), respectively. The temperature of the shortest crystallization time for the samples was observed at 105 °C. The overall isothermal melt crystallization kinetics of the three samples were further studied using the Avrami theory. Neat PLA showed a higher half-time of crystallization than that of the PLA/PCL and PLA/PCL/LACL blends, whereas the half-time of crystallization of PLA/PCL and PLA/PCL/LACL showed no significant difference. The addition of PCL decreased the spherulite size of crystallized PLA, and the nuclei density in the PLA/PCL/LACL blend was much higher than that of the PLA and PLA/PCL samples, indicating that LACL had a compatibilization effect on the immiscible PLA/PCL blend, thereby promoting the nucleation of PLA. The spherulites in the PLA/PCL and PLA/PCL/LACL blend exhibited a smeared and rough morphology, which can be attributed to the fact that PCL molecules migrated to the PLA spherulitic surface during the crystallization of PLA.

## 1. Introduction

Polylactide (PLA) is a thermoplastic aliphatic polyester, known as “green plastic” due to its renewable, biodegradable, and biocompatible characteristics [1,2,3]. Even if PLA has been frequently used in the biomedical industry and pharmaceutical fields, its broader application as a large-scale commodity and engineering material is still limited by its brittleness, slow crystallization rate, and low thermal stability [4,5,6,7]. To improve the toughness and crystallization kinetics of PLA, common routes including polymer blending, copolymerization, incorporation of nanofillers, and surface modifications have been developed [8,9,10]. Among these strategies, blending PLA with other polymers is the most versatile and economical method in industrial settings.

Poly(ε-caprolactone) (PCL) is also a biocompatible and biodegradable polyester, which exhibits a relatively low glass transition temperature (about −60 °C), a low melting point (about 60 °C), and high flexibility with an elongation at break of about 600% at room temperature [11,12,13]. Blends of PLA with PCL has been extensively studied, as PCL could improve the toughness of PLA and retain its biodegradability. However, many studies have reported that there are no favorable interactions between the two polymers [14]. Zhang et al. [15] prepared PCL/PLA, PEO/PLA, and PEG/PLA double-layer films to investigate the influence of the covered thin polymer layer on the spherulitic growth rates of PLA. Observation using phase contrast optical microscopy showed that the PCL/PLA pair was immiscible, which was responsible for the slower spherulitic growth rate of PLA than that of the PEO/PLA and PEG/PLA pairs. Since the mechanical properties of polymer blends are strongly influenced by the compatibility between the components, compatibilization is necessary for the immiscible PLA/PCL blends. A great amount of effort has been focused on incorporating compatibilizers to improve the interfacial adhesion of the blends. Chee et al. [16] added glycidyl methacrylate (GMA) as a reactive compatibilizer to improve the interfacial adhesion between PLA and PCL. The blends showed remarkable improved elongation at break and impact strength, and finer dispersion and smooth surface of the specimens were noted as GMA loading increased, indicating that the addition of GMA improved the interfacial compatibility of the immiscible blend.

It is also known that the mechanical properties of immiscible or partially miscible blends are greatly dependent on their solid-state morphology and crystallization behavior. Therefore, the study of the crystallization and morphology of PLA/PCL blends has received great interest. Rizzuto et al. [17] introduced two kinds of poly(lactide-*ran*-caprolactone) P(LA-*ran*-CL) random copolymers into the PLA/PCL (80/20) blends and paid attention to the spherulitic growth kinetics and overall isothermal crystallization kinetics of the PLA phase. They found that the copolymers induced plasticization effects that increased the crystallization ability of the PLA phase. The copolymer with the higher amount of ε-caprolactone and a lower *T*_g_ produced a larger plasticization effect and significantly increased the overall crystallization rate of PLA. The cold crystallization of PLA within the PLA/PCL (80/20) blends, with or without the addition of three types of poly(l-lactide-*block*-carbonate) (PLA-*b*-PC) diblock copolymers, was also investigated by the same group [18]. They found that the miscibility between PLA and PCL was improved with a reduced PCL droplet size when the copolymers were introduced. Using a 50/50 PLA-*b*-PC copolymer caused a threefold reduction in PCL particle size and a *T*_g_ depression of 10 °C for the PLA phase. They concluded that the crystallization of PCL droplets can accelerate the cold crystallization of PLA with enhanced nucleation. They also concluded that no significant nucleation effects were detected for the PLA/PCL blends during the melt crystallization. 

In addition, forming inclusion complexed compounds of PCL, PLA, and PCL-*b*-PLA results in highly suppressed microphase-separation and thus a compatible PLA/PCL blend and blocks in PCL-*b*-PLA copolymer, which are normally incompatible, as described above [19,20,21,22,23,24]. After removing the host and coalescence, the coalesced PCL/PLLA blend or PLC-*b*-PLLA showed no apparent crystallinity [20,21,22]. On the other hand, the inclusion of complexed PLLA with high stereoregularity and/or their diblock copolymer (PCL-*b*-PLA) with relatively low molecular weights revealed a relatively fast crystallization rate, with a short time of 20–140 s for complete crystallization [19]. However, enhancing mechanical properties for practical applications requires a high molecular weight, which in turns leads to a weakened crystallization rate due to long polymer chains and entanglement. Furthermore, most commercial PLLA has low optical purity due to its d-isomer acting as stereo defective parts, leading to further retarded crystallization ability. A l-Lactide-caprolactone copolymer (LACL) was employed as a compatibilizer in PLA/PCL blends in our previous study [25]. The effect of LACL on the morphology, mechanical properties, and cold crystallization behaviors of the blends was investigated. The addition of LACL decreased the dimensions of the dispersed PCL phase and PLA therefore crystallized at a lower temperature during the non-isothermal cold crystallization. The crystallization rate of PLA was accelerated, and the size of the crystals decreased with the addition of LACL during isothermal cold crystallization. Despite the reported crystallization behavior of the PLA/PCL blend [17,26], the effect of a small amount of random copolymer of l-Lactide-caprolactone on the melt crystallization kinetics, crystalline morphology, crystallinity as well as melting behavior has not yet been fully explored.

To further illustrate the compatibilization effect of LACL, its impact on the isothermal melt crystallization (i.e., the crystallization of PLA cooling from the melt) of high-molecular-weight PLA within PLA/PCL/LACL blends is investigated in this study. The isothermal melt crystallization kinetics and crystalline morphologies of PLA, PLA/PCL, and PLA/PCL/LACL are studied using differential scanning calorimeter (DSC) and polarized optical microscopy (POM). The related melting behavior and degree of crystallinity of crystallized samples were also investigated.

## 2. Materials and Methods

### 2.1. Materials

Polylactide (PLA, 2002D) with a high weight-average molecular weight (*M*_w_) of 2.6 × 10^5^ g/mol, a commercial product of NatureWorks Co. Ltd., Blair, NE, USA, had a d-isomer content of 4.25 wt %, a residual monomer content of 0.3 wt %, and a density of 1.24 g/cm^3^ [27,28,29]. The poly(ε-caprolactone) (PCL, CAPA6500 with a *M*_w_ of 8.5 × 10^4^ g/mol and a polydispersity index of 1.8) used in this study was purchased from Solvay Co. Ltd., Brussels, Belgium. Its melt flow index (MFI) was about 7 g/10 min (160 °C/2.16 Kg, ASTMD1238), and its –OH value was lower than 2 mg KOH/g. The lactide-caprolactone copolymer (LACL, PLC 7015) was kindly donated by Corbion Purac, Gorinchem, Netherlands. The molar ratio of the l-lactide/caprolactone is 70/30, and its *M*_w_ is 2.0 × 10^5^ g/mol.

### 2.2. Sample Preparation

PLA and PCL, in the form of pellets, and LACL in the form of powder, were separately dried in a vacuum oven at 80, 60, and 60 °C for 8 h, respectively. A PLA/PCL (80/20, *w*/*w*) blend and a PLA/PCL/LACL (80/20/5, *w*/*w*/*w*) blend were prepared through solution mixing and subsequent solvent casting. The blends were dissolved in chloroform and stirred at room temperature for 12 h to form 0.1 g/mL solutions. Then, the solutions were cast in Petri dishes, followed by solvent evaporation at room temperature for 24 h to form blend films. To remove residual solvent, the films were further vacuum dried at 60 °C for 8 h. The neat PLA was also prepared using the same procedure.

### 2.3. Characterization

The isothermal melt crystallization behavior of the samples was characterized using a TA Instru-ments Q20 DSC with Universal Analysis 2000 software. The samples were first heated from room temperature to 200 °C at a rate of 10 °C/min, and held for 3 min to eliminate any prior thermal history. Then, the samples were cooled to a chosen crystallization temperature (*T*_c_), in the range of 90 and 120 °C, at a rate of 60 °C/min (to avoid PLA crystallization during cooling), and held at *T*_c_ for a period of time until the crystallization was complete. The evolution of heat flow with crystallization time was recorded during the melt crystallization process for later data analysis. After isothermal melt crystallization at *T*_c_, the samples were heated up again to 200 °C at 10 °C/min to study their melting behavior. All experiments were performed under a nitrogen atmosphere.

The crystalline morphology of PLA, PLA/PCL, and PLA/PCL/LACL after isothermal melt crystallization at 95, 105, and 115 °C was studied using a POM (Olympus BX51, Olympus Corp., Tokyo, Japan) equipped with a charge coupled device (CCD) camera. The samples were sandwiched between two thin glass slides to a thickness of around 50 µm, heated to 200 °C on a hot stage, and then held for 3 min to eliminate any residual thermal history. Then, these molten films were promptly transferred to a preheated vacuum oven for crystallization at 95, 105, and 115 °C, respectively. The crystalline morphology of the samples was recorded using the CCD camera.

## 3. Results and Discussion

**Figure 1:**
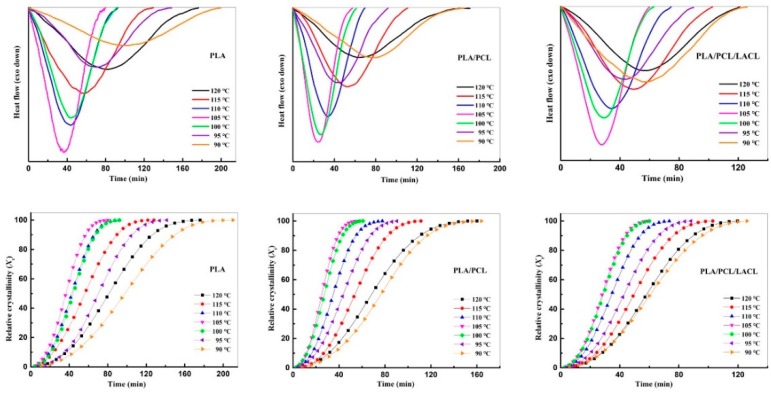
Heat flows and relative crystallinity as a function of time for PLA, PLA/PCL, and PLA/PCL/LACL isothermally melt crystallized at various temperatures characterized by differential scanning calorimeter (DSC).

Figure 1 shows the DSC heat flow and relative crystallinity (*X_t_*) as a function of time (*t*) for the samples isothermally melt crystallized at various temperatures (*T*_c_) between 90 and 120 °C. The *X_t_* as a function of *t* was calculated according to the following Equation (1):

(1) $$\;X_{t}=\frac{Q_{t}}{Q_{\infty }}=\frac{\int_{0}^{t}\left(\frac{dH}{dt}\right)dt}{\int_{0}^{\infty }\left(\frac{dH}{dt}\right)dt}\;$$

where *Q_t_* and *Q_∞_* are the amounts of heat generated at time *t* and infinite time, respectively, and d*H*/d*t* is the rate of heat evolution. From the figure, we can see that each sample exhibited only one exothermal peak with no secondary crystallization. Besides, for PLA, PLA/PCL, and PLA/PCL/LACL, the crystallization time for each sample decreased with increasing temperature up to 105 °C, indicating the enhanced mobility of PLA molecules upon obtaining much higher thermal energy and hence an enhanced crystallization rate. On the contrary, when the samples melt crystallized at temperatures higher than 105 °C, the crystallization time increased with increasing temperature, which indicates that the crystallization kinetics was suppressed with further increasing temperature. It is reasonable that the crystallization kinetics slowed down as supercooling decreased upon crystallizing at higher temperatures than the temperature (*T*_max_) of the maximum crystallization rate. Above *T*_max_, it became harder for the PLA molecules to align into crystalline regions, leading to weakened nucleation ability, which will be verified by the POM results. It should be noted that the shortest crystallization time (which corresponds to the maximum crystallization rate) for the samples crystallized from the glassy state (i.e*.*, cold crystallization) was seen at 120 °C in our previous work [25], indicating a different crystallization mechanism for melt crystallization and cold crystallization. 

The Avrami theory has been widely used to investigate the isothermal crystallization process for polymers [30]. According to the theory [31,32], the relative crystallinity (*X_t_*) develops with crystallization time (*t*), as

1 − *X_t_* = exp(−*kt^n^*) (2)

**Figure 2:**
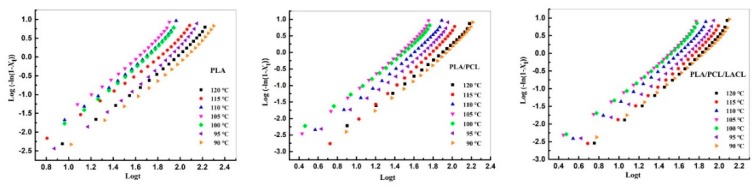
Avrami plots of PLA, PLA/PCL, and PLA/PCL/LACL isothermally melt crystallized at various temperatures.

The linear form of Equation (2) can be expressed as

log [−ln (1 −*X_t_*)] = log *k* + *n* log *t* (3)

where *n* is the Avrami exponent, which is dependent on the nature of nucleation and growth geometry of the crystals, and *k* is the overall rate constant associated with both nucleation and growth contributions. The overall isothermal melt crystallization kinetics of PLA, PLA/PCL, and PLA/PCL/LACL were calculated using Equation (3), and the corresponding Avrami plots are presented in Figure 2. The parameters *n* and *k* were obtained from the slopes and interceptions of the Avrami plots, respectively, and are summarized in Table 1.

It can be found that the values of *n* for PLA were between 2.14 and 2.42, indicating that the melt crystallization mechanism of PLA corresponded to three-dimensional spherulitic growth and heterogeneous nucleation. In previous work, the values of *n* for PLA were between 1.99 and 2.18 during isothermal cold crystallization, indicating that most of the crystals grew in two directions when PLA was heated from the glassy state. As shown in Table 1, the PLA/PCL blend showed slightly higher *n* values between 2.23 and 2.67, indicating that the addition of PCL did not change the crystallization mechanism and the geometry of the crystal growth of PLA. As compared to that of neat PLA, the PLA/PCL/LACL blend exhibited a narrowed range of *n* values between 2.17 and 2.38, which was also smaller than those of the PLA/PCL blend. This phenomenon further indicates that the addition of LACL had a compatibilization effect on the immiscible PLA/PCL blend. In addition, the *k* value for the samples all increased with the crystallization temperature *T*_c_, and then decreased with *T*_c_, after reaching a maximum value at 105 °C. It is inappropriate to directly compare the overall crystallization rate from the *k* values, because the unit of *k* is min^-*n*^ and *n* is not constant at different *T*_c_. Thus, the crystallization half-time (*t*_1/2_), which is the time required to achieve 50% of the final crystallinity of the samples, was introduced for an accurate evaluation of crystallization kinetics. The value of *t*_1/2_ was calculated using Equation (4).

(4) $$\;t_{1/2}={\left(\frac{\ln2}{k}\right)}^{1/n}\;$$

**Figure 3:**
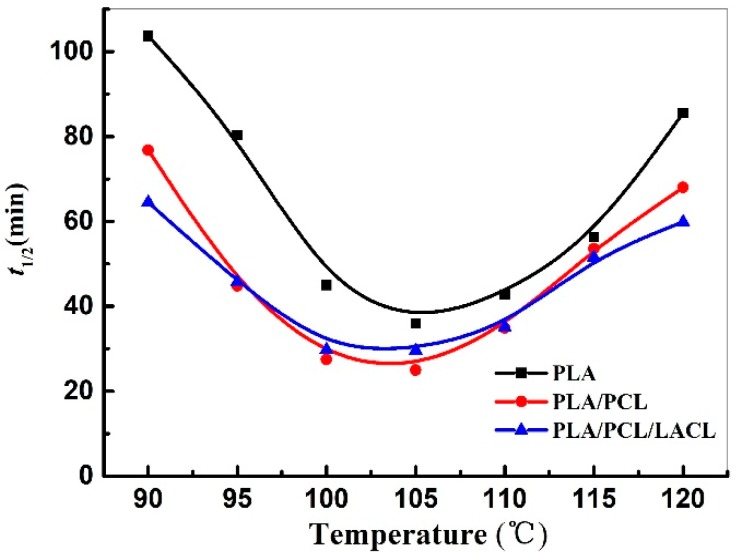
Crystallization half-time (*t*_1/2_) of PLA, PLA/PCL, and PLA/PCL/LACL isothermally melt crystallized at various temperatures.

The overall crystallization rates of the samples can be easily interpreted by comparing their *t*_1/2_. The variations of *t*_1/2_ for PLA, PLA/PCL, and PLA/PCL/LACL isothermally melt crystallized at different *T*_c_ are listed in Table 1 and presented in Figure 3. The *t*_1/2_ value for all the samples first decreased with increasing *T*_c_, reached the shortest crystallization time at 105 °C, and then increased with increasing *T*_c_. At a given *T*_c_, PLA showed a higher *t*_1/2_ value than that of the PLA/PCL and PLA/PCL/LACL blends, indicating that PCL accelerated the crystallization rate of PLA. However, the *t*_1/2_ value of PLA/PCL and PLA/PCL/LACL showed no significant difference, which indicates that further addition of LACL had little effect on the melt crystallization rate of PLA. As for the samples isothermally cold crystallized at the same temperatures in our previous study, neat PLA showed the fastest crystallization at 120 °C, while PLA/PCL and PLA/PCL/LACL exhibited the highest crystallization rate at 110 and 115 °C, respectively. The cold crystallization rate of PLA was accelerated by PCL, and further accelerated by LACL. On the other hand, compared to the *t*_1/2_ value (2–26 min) of cold crystallization in blends, the *t*_1/2_ values are 25–77 min for melt crystallization, indicating that the acceleration effects of PCL and PCL/LACL on the crystallization of PLA were greatly hindered during melt crystallization. 

These different crystallization behaviors for melt crystallization and cold crystallization may result from the different initial states prior to crystallization processes. For the cold crystallization process, PLA was heated from the glassy state, during which PCL remained in the crystalline state. When the increased temperature was beyond the melting point of PCL, the crystallization of PLA molecules was influenced by the melted PCL molecules. For example, in the PLLA/PCL blend, annealing the blends below the *T*_g_ (close to the *T*_m_ of PCL) of PLLA promoted nucleation and crystallization, resulting in decreased cold crystallization temperatures [18,26]. Particularly, annealing at lower temperatures, which favors the crystallization of PCL, caused more PCL crystals and thus a more apparent promotion effect on PLLA crystallization [18]. This effect arose from the potential nuclei of the PCL crystals upon heating from the glassy state of PLLA [18,26]. As such, during the cold crystallization of PLA/PCL and PLA/PCL/LACL in our previous work, the nucleation effect and nucleation density are much higher than was the case in the present work, as will be shown in the POM results. However, for the melt crystallization in this work, PLA was cooled from the melt. PCL always remained in the melting state throughout the whole crystallization process in the studied temperature range, providing no PCL crystals and thus no apparent nucleation effect induced by PCL. As such, the *t*_1/2_ value (25–77 min) of melt crystallization in blends was 3–12 times that (2–26 min) of cold crystallization as described above.

**Figure 4:**
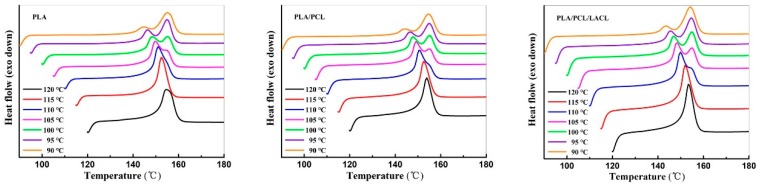
DSC heating scans of PLA, PLA/PCL, and PLA/PCL/LACL after isothermal crystallization at various temperatures.

Figure 4 presents the DSC heating scans of PLA, PLA/PCL, and PLA/PCL/ LACL which were isothermally melt crystallized at various temperatures. For the samples crystallized at relatively lower temperatures ranging from 90 to 105 °C, the DSC curves exhibited a double melting behavior. The two endothermic peaks corresponded to the melting crystals with different stability and perfection. When the samples crystallized at a lower *T*_c_, the higher degree of supercooling led to fast nucleation, which formed imperfect crystals with lower stability. With the increase in temperature during DSC heating, the crystals with lower stability first melted and were accompanied by recrystallization, which formed the first melting peak at the lower temperature. The second melting peak at the higher temperature corresponded to the melting of more perfect crystals created during the recrystallization process. This explanation is consistent with a melting-recrystallization origin revealed by temperature-modulated DSC in crystallized PLA with a d-LA content of ca. 6% in a previous study [33]. Accordingly, the second endothermic peak almost remained at a constant temperature of around 155 °C. With increasing *T*_c_, the formed crystals became more stable with a higher perfection and larger thickness, which resulted in a higher melting point according to the Gibbs−Thomson equation. Therefore, the first melting peaks gradually moved to a higher temperature and merged with the second melting peak. When the samples crystallized at a higher *T*_c_, the degree of supercooling was relatively low and the nucleation was accordingly slow, which enabled the crystals to have enough time to form stable crystals. Thus, a single melting peak was observed, which corresponded to the melting of the stable crystals. A single melting peak was observed for the samples after isothermal melt crystallization at 115 and 120 °C. For the samples isothermally melt crystallized at 110 °C, a single melting peak with a very tiny shoulder was detected in neat PLA, whereas the shoulder peaks were more apparent in the PLA/PCL and PLA/PCL/LACL blends, which was related to the enhanced chain mobility (and thus the enhanced crystallization/nucleation rate, as verified by the reduced *t*_1/2_ shown in Figure 3) of PLA influenced by PCL and LACL in the blend. Besides, the endothermic areas of the second melting peak for the PLA/PCL/LACL blend at various *T*_c_ were larger than those of the neat PLA and PLA/PCL blend, which indicates that the PLA chain mobility in the PLA/PCL/LACL blend was more active than that in the PLA/PCL, which was also attributed to the compatibilization effect of LACL between PLA and PCL. The crystallinity values of the samples after isothermal crystallization at various temperatures were calculated from the melting enthalpy and are listed in Table 2. Since only PLA molecules can melt at around 150 °C, the crystallinity calculated was from the PLA component in the samples. From Table 2, it is obvious that the crystallinity of all the samples increased gradually with increased isothermal crystallization temperature. Moreover, the crystallinity of PLA/PCL/LACL was higher than that of PLA/PCL and PLA after isothermal crystallization at each temperature, indicating that the chain mobility of PLA molecules was enhanced by PCL, further improved by incorporating LACL, finally leading to increased crystallinity of PLA in blends.

**Figure 5:**
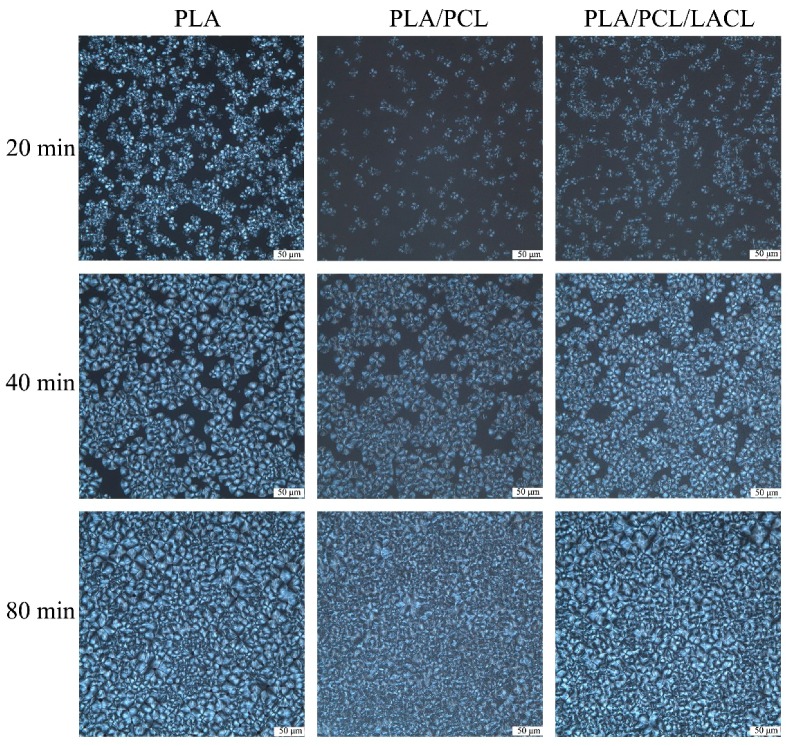
Polarized optical micrographs of PLA, PLA/PCL, and PLA/PCL/LACL spherulites isothermally melt crystallized at 105 °C for 20, 40, and 80 min.

Observing spherulite formation through an optical microscope allows us to trace the crystallization behavior of the samples. Figure 5 shows the POM micrographs of PLA, PLA/PCL, and PLA/PCL/LACL isothermally melt crystallized at 105 °C for different times. The samples formed well-defined spherulites that exhibited the classical Maltese-cross extinction pattern, as observed from the POM micrographs at 20 min. The size of the spherulites increased with the crystallization time. The addition of PCL increased the nuclei density, and as a result the spherulite size became smaller than that of the neat PLA. The reason for the increased nuclei density was that the addition of PCL increased the molecular chain mobility of PLA, which promoted the nucleation ability of PLA. The nuclei density in the PLA/PCL/LACL blend was much higher than that of the PLA and PLA/PCL samples, indicating that LACL had a compatibilization effect between PLA and PCL, and promoted the nucleation of PLA. As a result, the spherulites were prone to impinge on their neighbors to hinder further growth, resulting in smaller spherulites. Thus, the spherulite morphology of the samples was rough and it was hard to identify the Maltese-cross extinction pattern at 80 min.

**Figure 6:**
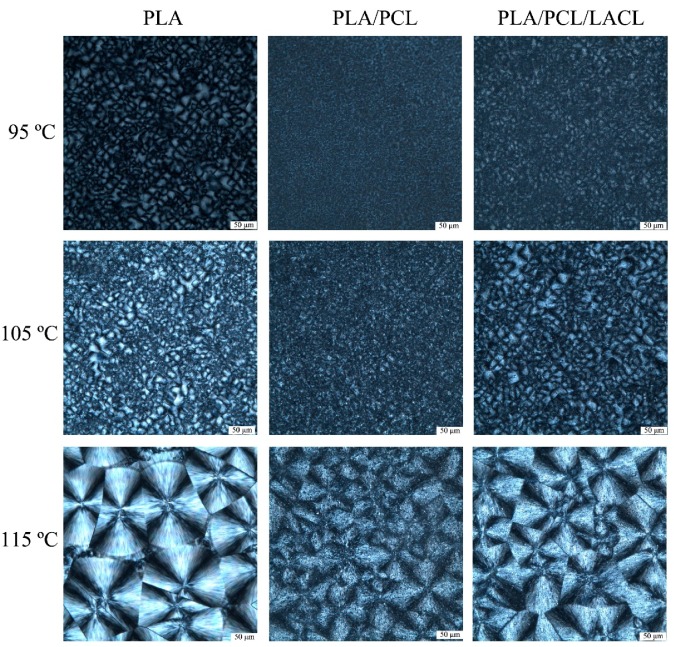
Polarized optical micrographs of PLA, PLA/PCL, and PLA/PCL/LACL spherulites isothermally melt crystallized at 95, 105, and 115 °C.

The POM micrographs of PLA, PLA/PCL, and PLA/PCL/LACL isothermally melt crystallized at 95, 105, and 115 °C are displayed in Figure 6. It is obvious that the size of the spherulites increased with the increasing crystallization temperature due to the decrease in nucleation density. When the samples melt crystallized at lower temperature of 95 and 105 °C, the nuclei density was too high to observe the spherulites with Maltese-cross morphology. The nuclei density of the spherulites in the PLA/PCL and PLA/PCL/LACL blend was higher than that of PLA in the whole temperature range, which indicates that the addition of PCL promoted the nucleation of PLA. For neat PLA isothermally melt crystallized at 115 °C, spherulites with a typical Maltese-cross extinction pattern were formed. However, in the PLA/PCL blend, the spherulites exhibited a smeared and rough morphology, and their size was smaller than that of PLA. This is associated with the fact that PCL molecules migrated to the PLA spherulitic surface during the crystallization of PLA. For the PLA/PCL/LACL blend, the spherulites also showed a rough morphology, but much clearer than that of PLA/PCL.

## 4. Conclusions

The isothermal melt crystallization behavior and crystalline morphology of PLA, PLA/PCL, and PLA/PCL/LACL were studied with DSC and POM. The crystallization time for the samples decreased with increasing temperature up to 105 °C. When the samples melt crystallized at temperatures higher than 105 °C, the crystallization time increased with increasing temperature. The overall isothermal melt crystallization kinetics of PLA, PLA/PCL, and PLA/PCL/LACL were studied using the Avrami theory. The melt crystallization mechanism of PLA corresponded to three-dimensional spherulitic growth and heterogeneous nucleation, and the addition of PCL and LACL did not change the crystallization mechanism and the geometry of the crystal growth of PLA. The addition of PCL accelerated the crystallization rate of PLA, but the further addition of LACL had little effect on the melt crystallization rate of PLA. The crystallinity of PLA was enhanced by PCL, and further improved by incorporating LACL. The addition of PCL increased the nuclei density and decreased the spherulite size of PLA. The nuclei density in the PLA/PCL/LACL blend was much higher than that of the PLA and PLA/PCL samples, indicating that LACL had a compatibilization effect between PLA and PCL, and promoted the nucleation of PLA. The spherulites in the PLA/PCL and PLA/PCL/LACL blend exhibited a smeared and rough morphology, and the size was smaller than that of PLA, which was because PCL molecules migrated to the PLA spherulitic surface during the crystallization process.

## Figures and Tables

**Table 1:** Avrami parameters of PLA, PLA/PCL, and PLA/PCL/LACL blends isothermally melt crystallized at various temperatures.

Samples	*T*_c_ (°C)	(*n*)	*K* (min^−*n*^)	*t*_1/2_ (min)
PLA	90	2.20	2.57 × 10^−5^	103.7
	95	2.26	3.45 × 10^−5^	80.3
	100	2.41	7.07 × 10^−5^	45.0
	105	2.42	1.22 × 10^−4^	35.9
	110	2.35	1.02 × 10^−4^	42.8
	115	2.14	1.22 × 10^−4^	56.3
	120	2.19	4.00 × 10^−5^	85.6
PLA/PCL	90	2.38	2.29 × 10^−5^	76.8
	95	2.26	1.29 × 10^−4^	44.8
	100	2.23	4.29 × 10^−4^	27.5
	105	2.47	2.42 × 10^−4^	25.0
	110	2.43	1.24 × 10^−4^	34.9
	115	2.67	1.64 × 10^−5^	53.6
	120	2.37	3.17 × 10^−5^	68.0
PLA/PCL/LACL	90	2.17	8.27 × 10^−5^	64.5
	95	2.22	1.45 × 10^−4^	45.8
	100	2.19	4.04 × 10^−4^	29.8
	105	2.15	4.77 × 10^−4^	29.6
	110	2.28	2.09 × 10^−4^	35.1
	115	2.38	5.83 × 10^−5^	51.6
	120	2.38	4.03 × 10^−5^	59.9

**Table 2:** Melting enthalpy and crystallinity of PLA, PLA/PCL and PLA/PCL/LACL after isothermal crystallization at various temperatures.

Samples	*T*_c_ (°C)	△*H* (J∙g^−1^)	*χ* ^α^
PLA	90	23.4	25.2%
	95	24.4	26.3%
	100	24.8	26.6%
	105	25.8	27.8%
	110	27.0	29.0%
	115	27.2	29.2%
	120	29.3	31.5%
PLA/PCL	90	19.5	26.2%
	95	20.2	27.2%
	100	20.6	27.7%
	105	21.1	28.4%
	110	22.1	29.7%
	115	23.9	32.1%
	120	24.7	33.2%
PLA/PCL/LACL	90	19.5	27.5%
	95	20.4	28.8%
	100	20.8	29.3%
	105	21.5	30.3%
	110	21.8	30.7%
	115	24.1	33.9%
	120	25.0	35.3%

^α^ Crystallinity *χ* = (∆*H*/∆H*)/Φ_PLA_; △*H* is the melting enthalpy calculated by integrating the melting peak in the DSC heating shown in Figure 4; ∆H* = 93 J∙g^−1^ is the melting enthalpy of a 100% crystalline PLA; and Φ_PLA_ is the weight fraction of PLA in the samples.
